# Editorial: Charged Particles in Oncology

**DOI:** 10.3389/fonc.2017.00301

**Published:** 2017-12-08

**Authors:** Marco Durante, Francis A. Cucinotta, Jay S. Loeffler

**Affiliations:** ^1^Trento Institute for Fundamental Physics and Applications (TIFPA), National Institute of Nuclear Physics (INFN), University of Trento, Rome, Italy; ^2^University of Nevada, Las Vegas, NV, United States; ^3^Massachusetts General Hospital, Harvard Medical School, Boston, MA, United States

**Keywords:** radiotherapy, particle therapy, proton therapy, carbon ions, space radiation

**Editorial on the Research Topic**

**Charged Particles in Oncology**

High-energy charged particles represent a cutting-edge technique in radiation oncology (1). Protons and carbon ions are used in several centers all over the world for the treatment of different solid tumors. Typical indications are ocular malignancies, tumors of the base of the skull, hepatocellular carcinomas, and various sarcomas (2). The physical characteristics of the charged particles (Bragg peak) allow sparing of much more normal tissues than it is possible using conventional X-rays (3), and for this reason, all pediatric tumors are considered eligible for proton therapy (4). Ions heavier than protons also display special radiobiological characteristics, which make them effective against radioresistant and hypoxic tumors (5).

Protons and ions with high charge (Z) and energy (HZE particles) represent a major risk for human space exploration (6, 7). The main late effect of radiation exposure is cancer induction (8), and at the moment the dose limits for astronauts are based on lifetime cancer mortality risk (9, 10). The Mars Science Laboratory measured the dose on the route to Mars (11) and on the planet’s surface (12), supporting predictions that a human exploration mission to Mars will exceed the radiation risk limits (7, 13). Notwithstanding many studies on carcinogenesis induced by protons and heavy ions, the risk uncertainty remains high with important risk assessment questions to non-targeted effects (13) and the “quality” of HZE particle-induced tumors compared to spontaneous and photon-induced tumors (8).

In this research topic, we invited scientists studying high-energy charged particles either for cancer treatment or for space radiation protection. We believe that space radiation protection and particle therapy share many common problems, and this research topic can be an inspiration to find applications and answers from fields that are apparently far away. Physics, biology, and medical contributions in this field will be found in the volume, owing to the fact that the field of charged particles in oncology is highly interdisciplinary.

The research topics accepted 59 articles including a total of 351 authors, demonstrating the high interest in this field. It is in the top five most viewed research topics of this journal. The articles can be divided into the following topics.

## Physics

A number of contributions deal with medical physics in particle therapy. Articles with most views are the reviews of range verification methods in particle therapy by Aafke Kraan and of the FLUKA Monte Carlo code. FLUKA is widely used at CERN but, from high-energy physics uses, has found several applications both in particle therapy and space radiation simulations. Cary Zeitlin and Chiara La Tessa describe the role of nuclear fragmentation in particle therapy and space radiation shielding. A highly viewed article by Kim et al. details how nuclear fragmentation can be exploited to simulate the full galactic cosmic ray spectra using a few particle beams with applications in biological countermeasure and radiation shielding studies. Six articles deal with experimental methods for range verification in particle therapy, using prompt γ-rays, secondary charged particles, 4D-PET, or ultrasounds. For space radiation protection, the use of active detectors for personal dosimetry is described, and potential applications of superconducting magnets for active shielding are reported from the SR2S European project.

## Biology

Several articles deal with experimental radiobiology and carcinogenesis induced by high-energy charged particles. In a highly viewed article, Sishc et al. discuss the role of telomeres in radiation-induced carcinogenesis. Cellular effects of charged particles in comparison to X-rays are reported in tumor cells and in normal human endothelial cells. Chromosome aberrations and DNA damage response pathways are the main topic of five different articles. Specific molecular pathways following exposure to charged particles are also described in various articles, e.g., transcription factors, prostaglandin (“Phoenix rising” effect), bone marrow and mammary cell reprogramming, β-catenin, proinflammatory signals.

## Medicine

Twelve manuscripts report clinical results of particle therapy. Schiller et al. review the results of particle therapy in prostate cancer, while two articles summarize the Japanese experience with carbon ion therapy in Chiba and Gunma. The articles on second cancers and late effects after proton therapy are important examples of the bridge between therapy and space: the results are, in fact, also very important for the assessment of late effects caused by cosmic rays in crews of long-term space missions. Lane et al. describe results with X-rays using hypofractionation, and how high dose/fractionation can be beneficial using protons and heavy ions. Modeling clinical results is described in other articles, dealing with RBE in proton therapy and combination of particle therapy and chemotherapy. An interesting article from Specht et al. collects the data on fast neutron therapy and indicates what can be learned form that experience in charged particle therapy. Fast neutrons are an important risk factor for space missions, too.

## Facilities and Networks

Finally, a few articles are dedicated to the analysis of facilities for ground-based space research and preclinical radiobiology, mini-beams in therapy, and networks and educational activities in particle therapy.

The high number of articles submitted, and their excellent quality, indicates that the topic is considered of great interest for researchers in many different fields. Looking at the overall picture, it is clear that space radiation protection and particle therapy share many common research problems (14) and can learn from each other. Differences are also clear: space radiation protection conditions are whole body, low-dose rate for a specialized group of highly skilled workers; radiotherapy is characterized by partial body, high-dose, fractionated exposures of cancer patients. Nevertheless, many research topics are similar: late effects (see Newhauser et al. in this issue), including cancer (see articles by Locke and Weil and Eaton et al. in this issue) and tissue degenerative effects such as cardiovascular (15) and CNS (16); individual radiosensitivity (see the article by Shim et al. in this article); particle and neutron dosimetry (see Schneider and Hälg in this issue) and microdosimetry (17); transport calculations with Monte Carlo (Lima et al. in this issue) and analytical codes; radioprotectors; and non-targeted effects (see the article by Barcellos-Hoff and Mao in this issue). They also share common platforms for research. We hope that this ebook will be useful for researchers working on charged particles in looking for answers to the many questions that these two topics, only apparently far away, share.

## Author Contributions

All authors contributed equally to this editorial.

## Conflict of Interest Statement

The authors declare that the research was conducted in the absence of any commercial or financial relationships that could be construed as a potential conflict of interest.
